# The distribution of blinkrate among Malawian young adults: a cross-sectional study

**DOI:** 10.1038/s41598-023-29016-x

**Published:** 2023-02-04

**Authors:** Mayamiko Mbamba, Thokozani Mzumara, Precious Chisale, Joseph Afonne

**Affiliations:** 1 Mzuzu University, Private Bag 201, Mzuzu, Malawi; 2 Mzuzu Central Hospital, Private Bag 209, Mzuzu, Malawi; 3 Mzimba-North District Hospital, P.O Box 299, Mzuzu, Malawi; 4 Department of Optometry, Mzuzu University, Private Bag 209, Luwinga, Mzuzu, Malawi

**Keywords:** Physical examination, Eye diseases

## Abstract

Blink rate is a critical sign for numerous systemic and ocular conditions in medicine, however, the literature reports varying values for the parameter. Hence, the aim of this study was to establish the cut-off blink rate value among Malawian young adults including the effects of sex and age on the parameter. This was a quantitative descriptive cross-sectional study conducted among students at Mzuzu University in Malawi. The study recruited 98 participants, 50% male and 50% female. The age ranged from 17–45 years. The blink rate was measured manually by observing the number of blinks per minute. The average blink rate was 16.04 (SD = 6.417) blinks per minute. The Blink rate was not significantly correlated with age (*P* = 0.066) and sex (*P* = 0.8143). Our study confirms that blink rate varies according to geographical location as a factor of different weather conditions. Moreover, we found no age and sex-related differences in blink rate.

## Introduction

Blinking is integral to the ocular surface integrity by distributing the pre-corneal tear film thus protecting the ocular surface from environmental hazards besides removal of ocular debris and foreign bodies^1,2^. Categorically, blinking is in two main forms, Voluntary and involuntary blinking^3^. Specifically, Voluntary blinking results from a decision to blink, while involuntary blinking can either be reflex, from automatic response to objects invading the eye, or spontaneous blinking, from perception of information processing^4^. Accordingly, Borsch defines spontaneous blinking as the automatic closure of eyelids without any external stimulation to systematically restore the tear film^5^.

The tear film is composed of three layers. The first layer, aqueous layer produced by the lacrimal gland, is rich in proteins and other substances. The second layer, the lipid layer produced by Meibomian glands, prevents tear evaporation. Lastly, the mucin layer produced by the goblet cells prevents friction on the corneal surface^6^. As a consequence, the blink pattern reflects the time the tear film remains intact following a blink^7^.

Physiologically, blinking involves contracting the antagonistic muscles of the levator palpebrae superioris and the orbicularis oculi alternatively in a push–pull fashion and is under the control of the cortical system^6^. As such, the blink rate is a significant indicator for both systemic and ocular conditions. Systemically, a low blink rate indicates hypodopaminergic activity (e.g. Parkinson disease, progressive supranuclear palsy and attention deficit/hyperactivity disorder), while a high blink rate is indicative of hyperdopaminergic activity (e.g. Huntington disease, schizophrenia, and neuro-developmental conditions)^6^. On the ocular surface, a low brink rate is a risk factor of dry eye due to tear evaporation^8^ and disruption of the tear film initiates ocular symptoms such as, discomfort, eye strain, burning sensation, excessive lacrimation, pain, visual disturbances^9^.

The normal spontaneous blink rate among humans is recorded between 6 and 30 blinks per minute attributed to a large inter individual difference^10^, and partly lack of unified measuring method. Clinically, diverse techniques measure spontaneous blink rate ranging from sophisticated procedures involving videoscope, and video camera to simple manual observation^11^. Needless to say, the gold standard of blink rate value is absolute^10^.

Apparently, many different factors such as, temperature, attention, air draught speed and lighting affect the blink rate^12^. Thus, dissimilar values for normal blink rate have been reported elsewhere^3^. Regardless, to the best of our knowledge there is lack of data on normative values of blink rate for the Malawian population. Given that blink rate can vary with climatic conditions and also measurement methods, therefore, this assessment aimed at providing healthcare practitioners with reference value of blink rate for this population measured using cheap and convenient equipment^13^. In addition, it evaluates age and gender differences of blink rate among the population group. The result of this study can aid clinical diagnosis of medical and ocular problems in low resource settings.

## Methods

We conducted a quantitative cross-sectional study at Mzuzu University, in Malawi. Our target population was students aged from 15–49 years of age. The study recruited a total of 98 participants. We employed stratified random sampling technique to select a proportion of student distribution per faculty, and within each stratum we nominated participants by simple random sampling method. The proportion per faculty was as follows: faculty of environmental science, 21 (22.2)%, faculty of education 56 (57.1%), faculty of health science 10 (10.4%), faculty of information science 7% (6.7%) and faculty of tourism 4 (3.6%).

We included all students aged above 15 years and below 49 years. As an exclusion criterion, we excluded all participants with ocular pathologies, ocular surface abnormalities, history of neurological diseases and contact lens use. Again, we excluded all participants on topical and systemic prescription medicine, and current cigarette smokers. Ametropic Participants or those wearing spectacles were also excluded.

The ethics committee of Mzuzu University Faculty of Health Sciences (clearance number: Fhsrc/17/0005) and the Mzuzu University Research Directorate endorsed our study. In addition, we adhered to the declaration of Helsinki as such; all subjects gave written informed consent; we maintained anonymity by utilizing codes for identification; and no participant was harmed during the study (Supplementary material).

### Procedures

We conducted the procedure in a standard optometric clinic consultation room, free from significant background noise. Before the test, we allowed each participant 5 min adaptation period as recommended for blink behavior analysis^6^. Thereafter, without providing prior information, we instructed participants to gaze at a target 3 m away while we manually noted the number of complete blinks per minute using a stop watch^1^. Before the study, a pilot study was conducted to check the validity of the data collection tool. During the study, in order to lessen observer errors incurred due to the objective nature of the procedure and ensure reliability, we repeated the procedure twice and considered the average as final blink rate value. Finally, we logged the final value onto a preform including participant’s demographic information, consisting of age and sex.

### Analysis

First, we got the data into Statistical Package for the Social Scientist (SPSS) version 16.0. Then, we operated Pearson correlation test to calculate the correlation between blinking rate and age, and student’s independent *t* test to compare the mean blink rate between sex. Next, we illustrated descriptive statistics and correlations with bar graphs, and scatterplots respectively. And where appropriate, we considered the value of *p* < 0.05 statistically significant.

## Results

The 98 participants encompassed 50% (49) females and the age ranged from 17 to 45 years (Table 1). The overall average blink rate was 16.04 (SD = 6.417) blinks per minute and ranged from 4 to 33 blinks.

**Table 1:** Distribution of study characteristics.

Sample characteristics	N	%
Gender		
Male	49	50.0
Female	49	50.0
Age group		
15–19	10	10.5
20–24	55	56.1
25–29	22	22.4
30–34	5	5.1
35–39	2	2.0
40–44	2	2.0
45–49	2	2.0

### Correlation between age and blink rate

**Figure 1:**
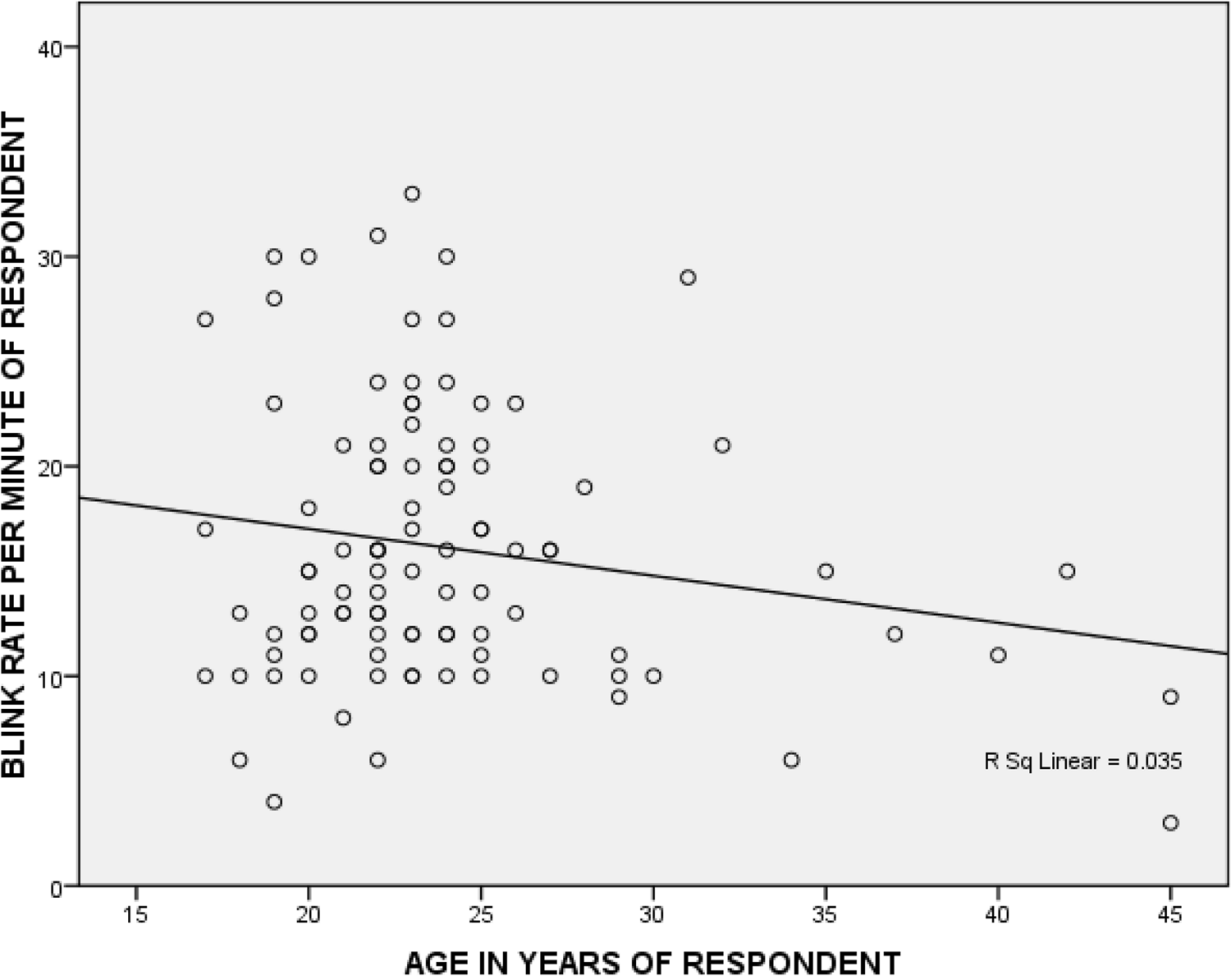
Correlation between age and blink rate.

The Pearson’s correlation test depicted a weak negative correlation between blink rate and age which was not statistically significant (*r* =  − 0.186, *r*^2^ = 0.035, *P* = 0.066). Figure 1.

### Distribution of blinking rate according to gender

**Figure 2:**
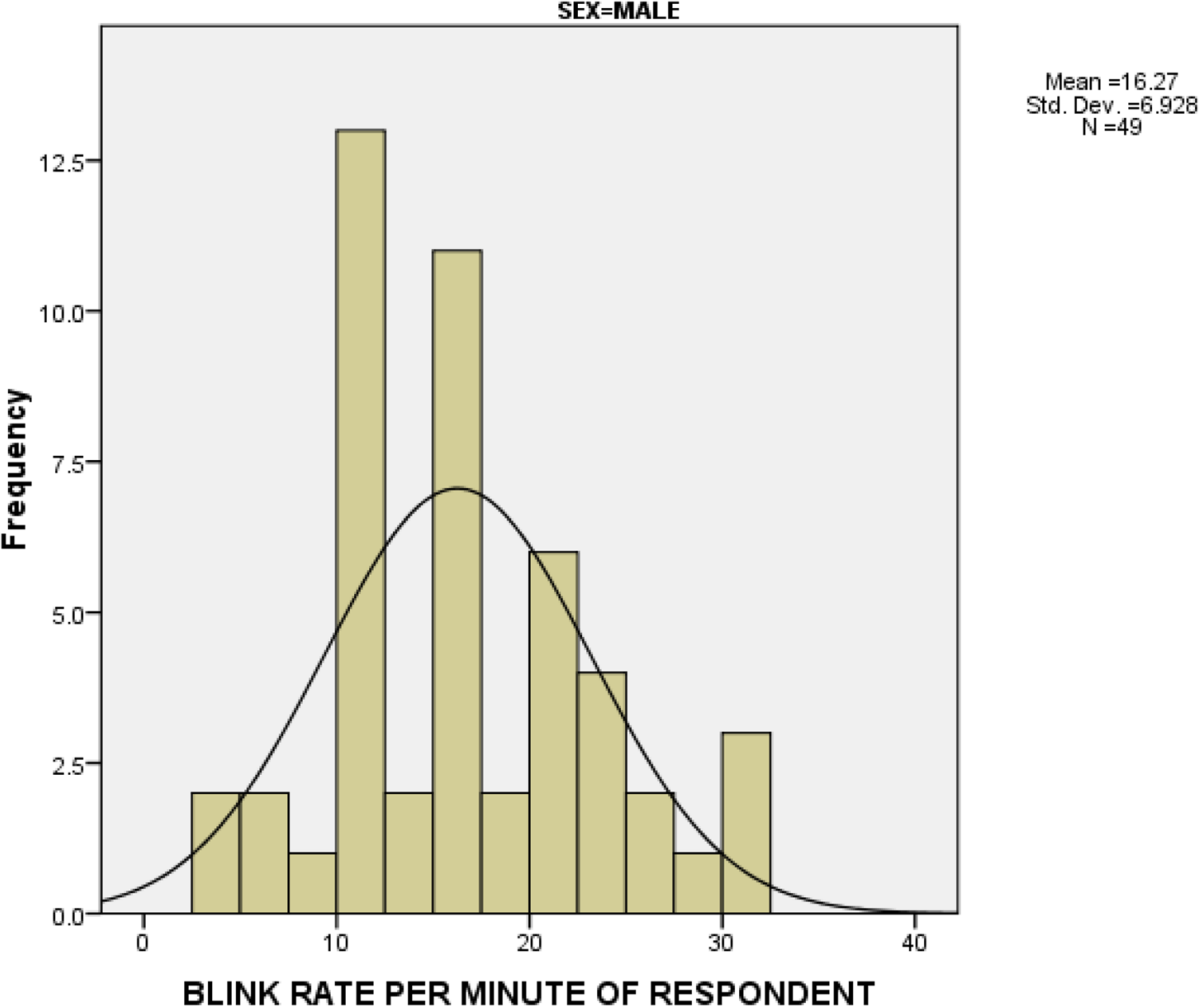
Distribution of blinking rate among males.

**Figure 3:**
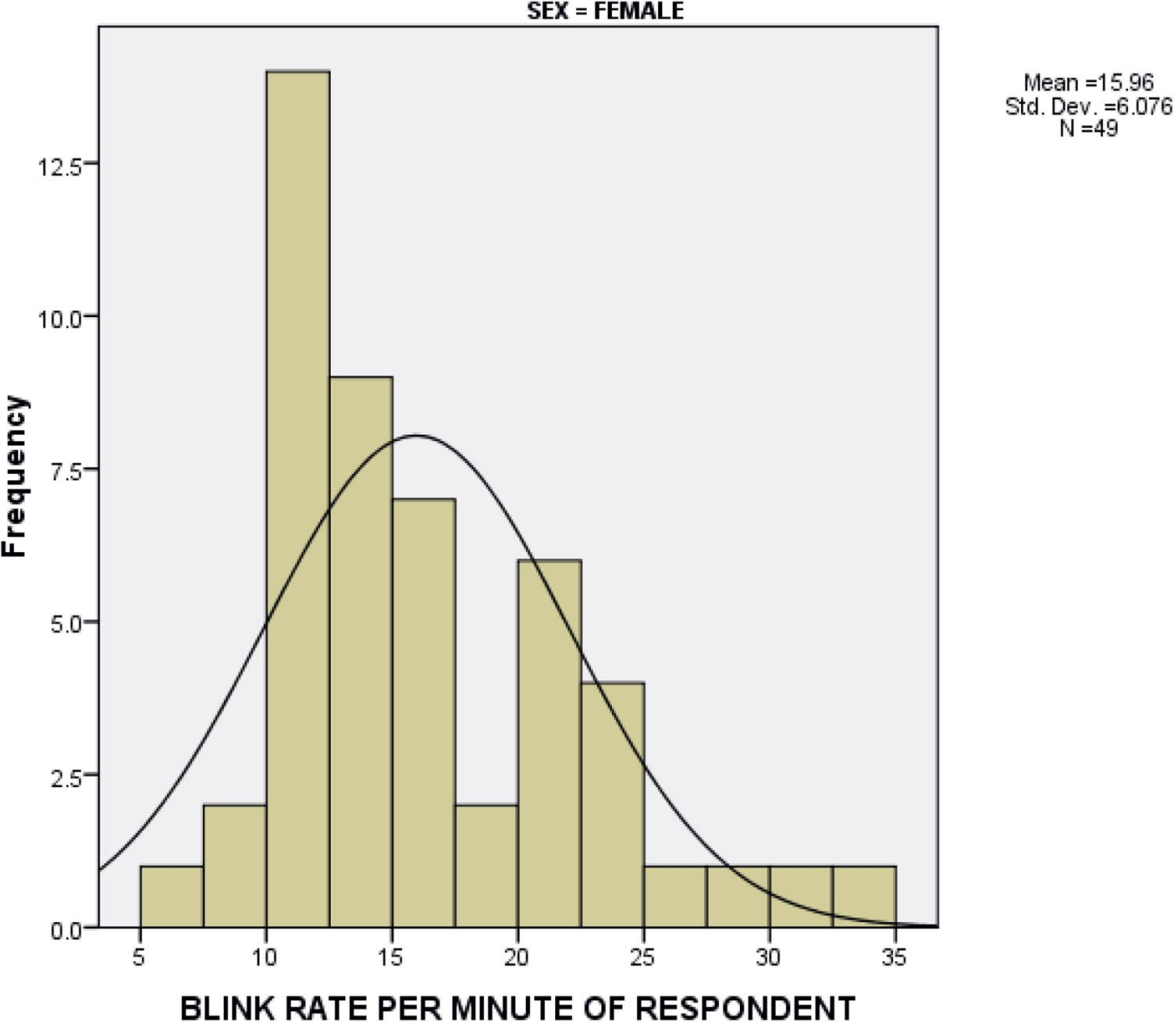
Distribution of blinking rate among females.

The average blinking rate was 16.27 ± 6.928 blinks/minute and 15.96 ± 6.076 blinks/minute among males and females respectively. An independent *t* test depicted that the mean difference between sex was not statistically significant. (*t* (96) = 0.2355, *P* = 0.8143). Figures 2 and 3.

## Discussion

In the current review, the average blink rate was 16.04 ± 6.417 blinks per minute, similar to Zametkin et al. (1979)^14^. Nevertheless, disparate blink rate values are reported in different settings. For instance, it was relatively lower (12.83 ± 7.64 blinks/min) in Switzerland^6^, and higher in Saudi Arabia, 19.74 ± 9.12 blinks/min, and Japan 18.2 ± 14.1 blinks/min^10^. The variation could be due to different weather conditions in the disparate areas understudy. According to Nakamori et al.^15^, humidity affects blinking rate because of increasing tear evaporation. And in part, it could also be due to different techniques employed for measuring blink rate^1,6^.

Likewise, previous studies^16–18^ the current study found no significant correlation between blink rate and age. Even though, sugiyama and colleagues^10^ in a study to establish normative data for different age groups using a wider age range reported a significant positive correlation between age and blink rate among infants but not older age groups. They concluded that from birth the neaonates blink infrequently and their blink rate increases stabilizing by age of 10 years after which no significant age related changes occur. The results of our study are unsurprising given the age range understudy (15–45). Further studies can focus on wider age group to further elucidate the phenomenon.

Regarding sex, the study found statistically non-significant difference in blink rate values, although females had slightly lower blink rate compared to males(P = 0.8143). This is in accord with Doughty^18^. On the contrary, Bentivoglio and colleagues^19^ found higher blinking rates among women than men. However, they evaluated blink rate in relation to behavioral tasks for instance when reading. The results of our study could be attributed to the nature of blink rate; Our study did not assess blink rate during reading activity. Despite sex related differences, mainly due to hormones in adnexal and ocular surface tissues including conjunctiva and lacrimal glands^20^, the larger inter-individual variability in blink rate disguises smaller gender effects^18^.

Our study was not without limitations. The main limitation was the environment understudy, which was not fully controlled as the results could be affected by daily temperature changes. Moreover, the study did not differentiate intentional and unintentional blinking which would have affected the results. Furthermore, the sample size was small and could be considered a national or regional representation. In addition, our age range was narrow, hence, we could not fully elucidate the effect of age on the younger and older age groups. The main strength of our study is that it offers a benchmark of blink rate for the Malawian population. Furthermore, our study uses cheap noninvasive appliances applicable in low resource settings. The results could be significant for future research or survey involving the external eye in Malawi.

In conclusion, the average blink rate among Malawian young adults is within the expected normal range, however, it is different from other settings. On average, blink rate was 16.04 (SD = 6.417) and was not related to age and sex. This confirms that blink rate varies according to climatic conditions. Further studies can include a wider age range to confirm these findings in other age groups. Again, other studies can assess the blink rate during different behavioral tasks/activities. The findings of the study provide critical insight regarding the blink behavior patterns of Malawians as a measure of individual engagement with scene content.

## Supplementary Information

Supplementary Information.

## Data Availability

The datasets generated and analyzed during the current study can be made available upon request through the corresponding author (JA).
