# Supplementary material for: The distribution of blinkrate among Malawian young adults: a cross-sectional study
Source: Sci Rep. 2023 Feb 4;13:2039. doi: 10.1038/s41598-023-29016-x (PMC9899223; doi:10.1038/s41598-023-29016-x)
Supplement: Supplementary file 1 — Supplementary Information. [file 41598_2023_29016_MOESM1_ESM.pdf]

Gender	Age	Blinkratepe	Medicatior	Surgery	Contactlen	Eyeproblem
female	20-24	20	no	no	no	no
male	15-19	28	no	no	no	no
male	25-29	21	no	no	no	no
male	25-29	11	no	no	no	no
male	35-39	15	no	no	no	no
male	20-24	12	no	no	no	no
female	15-19	10	no	no	no	no
female	15-19	6	no	no	no	yes
female	20-24	12	no	no	no	no
female	20-24	16	no	no	no	no
female	20-24	12	no	no	no	no
male	25-29	19	no	no	no	yes
male	20-24	21	no	no	no	no
male	25-29	14	no	no	no	no
male	25-29	11	no	no	no	no
female	30-34	29	no	no	no	no
female	25-29	16	no	no	no	no
male	20-24	30	no	no	no	no
male	20-24	16	no	no	no	no
male	25-29	16	no	no	no	no
female	20-24	8	no	no	no	no
female	20-24	10	no	no	no	no
female	20-24	14	no	no	no	no
female	20-24	15	no	no	no	no
female	20-24	33	no	no	no	no
female	20-24	11	no	no	no	no
male	30-34	10	no	no	no	no
male	25-29	10	no	no	no	no
male	25-29	19	no	no	no	no
female	20-24	20	no	no	no	no
male	25-29	10	no	no	no	no
female	20-24	16	no	no	no	no
female	20-24	16	no	no	no	no
female	25-29	10	no	no	no	no
female	20-24	14	no	no	no	no
female	20-24	12	no	no	no	no
female	15-19	10	no	no	no	no
female	20-24	24	no	no	no	no
female	30-34	21	no	no	no	no
female	20-24	10	no	no	no	yes
female	20-24	18	no	no	no	yes
female	15-19	17	no	no	no	no
female	20-24	23	no	no	no	no
female	20-24	18	no	no	no	no
female	20-24	22	no	no	no	no
female	25-29	17	no	no	no	no

female	20-24	14	no	no	no	no
female	20-24	20	no	no	no	no
female	40-44	11	no	no	no	no
female	35-39	12	no	no	no	no
female	20-24	13	no	no	no	no
female	20-24	23	no	no	no	no
female	20-24	20	no	no	no	no
female	20-24	13	no	no	no	no
female	20-24	30	no	no	no	no
female	20-24	10	no	no	no	no
female	15-19	12	no	no	no	no
female	20-24	13	no	no	no	no
female	15-19	27	no	no	no	yes
female	20-24	13	no	no	no	no
female	25-29	9	no	no	no	no
female	20-24	13	no	no	no	no
female	15-19	13	no	no	no	no
female	20-24	21	no	no	no	no
female	25-29	12	no	no	no	no
male	20-24	24	no	no	no	no
male	20-24	27	no	no	no	no
male	25-29	10	no	no	no	no
male	20-24	12	no	no	no	no
male	15-19	4	no	no	no	no
male	20-24	20	no	no	no	no
male	20-24	31	no	no	no	no
male	25-29	20	no	no	no	no
male	15-19	30	no	no	no	no
male	25-29	16	no	no	no	no
male	25-29	23	no	no	no	no
male	20-24	12	no	no	no	no
male	25-29	13	no	no	no	no
male	20-24	27	no	no	no	no
male	20-24	12	no	no	no	no
male	25-29	23	no	no	no	no
male	20-24	15	no	no	no	no
male	25-29	17	no	no	no	no
male	30-34	10	no	no	no	no
male	20-24	15	no	no	no	no
male	20-24	18	no	no	no	no
male	20-24	15	no	no	no	no
male	20-24	11	no	no	no	no
male	20-24	10	no	no	no	no
male	45-49	3	no	no	no	no
male	20-24	16	no	no	no	no
male	30-34	6	no	no	no	no
male	45-49	9	no	no	no	no

male	40-44	15	no	no	no	no
male	20-24	21	no	no	no	no
male	20-24	6	no	no	no	no
male	25-29	20	no	no	no	no
male	20-24	19	no	no	no	no
